# Autophagy as a Target for Non-Immune Intrinsic Functions of Programmed Cell Death-Ligand 1 in Cancer

**DOI:** 10.3390/ijms241915016

**Published:** 2023-10-09

**Authors:** Blanca Estela García-Pérez, Christian Pérez-Torres, Shantal Lizbeth Baltierra-Uribe, Juan Castillo-Cruz, Nayeli Shantal Castrejón-Jiménez

**Affiliations:** 1 Departmento de Microbiología, Escuela Nacional de Ciencias Biológicas, Instituto Politécnico Nacional, Prolongación de Carpio y Plan de Ayala S/N, Col. Santo Tomás, Alcaldía Miguel Hidalgo, Mexico City 11340, Mexico; 2 Departmento de Posgrado e Investigación, Escuela Superior de Medicina, Instituto Politécnico Nacional, Prolongación de Carpio y Plan de Ayala S/N, Col. Santo Tomás, Alcaldía Miguel Hidalgo, Mexico City 11340, Mexico; 3 Área Académica de Medicina Veterinaria y Zootecnia, Instituto de Ciencias Agropecuarias, Universidad Autónoma del Estado de Hidalgo, Av. Universidad km. 1. Exhacienda de Aquetzalpa A.P. 32, Tulancingo 43600, Mexico

**Keywords:** PD-L1, autophagy, mTOR, cancer, immunotherapy

## Abstract

Autophagy is a catabolic process that is essential to the maintenance of homeostasis through the cellular recycling of damaged organelles or misfolded proteins, which sustains energy balance. Additionally, autophagy plays a dual role in modulating the development and progression of cancer and inducing a survival strategy in tumoral cells. Programmed cell death-ligand 1 (PD-L1) modulates the immune response and is responsible for maintaining self-tolerance. Because tumor cells exploit the PD-L1–PD-1 interaction to subvert the immune response, immunotherapy has been developed based on the use of PD-L1-blocking antibodies. Recent evidence has suggested a bidirectional regulation between autophagy and PD-L1 molecule expression in tumor cells. Moreover, the research into the intrinsic properties of PD-L1 has highlighted new functions that are advantageous to tumor cells. The relationship between autophagy and PD-L1 is complex and still not fully understood; its effects can be context-dependent and might differ between tumoral cells. This review refines our understanding of the non-immune intrinsic functions of PD-L1 and its potential influence on autophagy, how these could allow the survival of tumor cells, and what this means for the efficacy of anti-PD-L1 therapeutic strategies.

## 1. Introduction

Autophagy is a complex and dynamic recycling system responsible for the removal of senescent or malfunctioning organelles. The cytoplasmic material sequestered via autophagy is delivered to lysosomes for degradation, allowing for its eventual reutilization. In recent years, it has been shown that this process is a relevant topic for researchers, as alterations in autophagy have been implicated in various infectious diseases, neurodegenerative diseases, metabolic diseases, and cancers, among other conditions [1,2,3]. In many diseases, autophagy seems to play a dual role; for this reason, its manipulation as a form of therapy continues to be controversial. As a catabolic pathway, autophagy maintains the quality control of cells. This homeostatic process removes damaged organelles, misfolded proteins, and microorganisms; additionally, it helps preserve the cell during starvation and oxidative stress [4]. These elements are degraded and recycled via the lysosome-dependent pathway. Paradoxically, this homeostatic cellular pathway plays a dual role in cancer; this role depends on the stage of cancer development, nutrient accessibility, microenvironmental stress, pathogenic conditions, and the immune response [5]. It has been demonstrated that autophagy suppresses chronic tissue damage, preventing tumor initiation, especially in the early stages of tumorigenesis. Moreover, autophagy contributes to the maintenance of genomic stability, the suppression of oxidative stress, and the inhibition of NRF2 activation, and it also plays a protective role by suppressing metastasis [6,7]. Different studies have demonstrated that the molecules involved in autophagy are the key to suppressing tumors. Beclin-1 is a protein that is involved in the early stages of autophagy and has been associated with being a tumor suppressor. Beclin-1 overexpression has been shown to suppress the progression of gastric cancer by promoting apoptosis and reducing cell migration [8]. Moreover, it is known that Beclin-1^−/−^ mutant mice die early in embryogenesis, and Beclin-1^+/−^ mutant mice suffer from a high incidence of spontaneous tumors [9]. Wijshake and colleagues found that Beclin-1 promotes the localization of E-cadherin on plasma membranes. Thus, Beclin-1, as a breast-tumor-suppressor molecule, restricts tumor growth and metastases when E-cadherin is present at the cell surface [10]. Another group of proteins involved in the autophagy process in relation to the inhibition of cancer progression are autophagy-related proteins (ATGs), which are involved in membrane dynamics during autophagy [11,12]. ATG proteins are responsible for orchestrating the autophagic process, which allows for the elimination of damaged proteins and organelles, cellular stress, and aging. A study showed that mice with a systemic mosaic deletion of ATG5 developed multiple benign adenomas only in the liver; the analysis of hepatic cells demonstrated the presence of abnormally enlarged mitochondria, the accumulation of reactive oxygen species, and genomic damage. Through an analysis of ubiquitin-positive aggregates and a higher accumulation of p62 (a selective substrate of autophagy), the authors confirmed the role of defective autophagy in the development of tumors. Moreover, they extended their study of this topic in a model of liver-specific ATG7-deficient mice, which confirmed that tumor progression was partially suppressed via p62 deletion [13]. This evidence emphasizes the role of autophagy and its related proteins as tumor-suppressing factors.

Regarding the other approach to autophagy in cancer cells, several studies have demonstrated that the autophagy pathway supplies tumor cells with nutrients [14,15], increases the survival of cancer cells and their resistance to stress factors [16], lowers p53 levels [17], favors tumor metabolism [18,19], inhibits the surface expression of MHC-I on cancer cells, protecting them from lymphocyte cytotoxicity [20], and promotes metastasis [21]. Moreover, an association between autophagy and programmed cell death-ligand 1 (PD-L1) expression has recently emerged. This relationship has captured the attention of researchers because the abnormally high expression of PD-L1 in tumor cells and antigen-presenting cells mediates the immune evasion of tumors. In physiological conditions, PD-L1 is a molecule that intricately regulates the immune response triggered by T lymphocytes based on recognition by PD-1 and plays an important role in the maintenance of self-tolerance [22]. Since the first description of immune checkpoint molecules, intensive research has been conducted, mainly because cancer cells take advantage of these molecules to deactivate the effector functions of lymphocytes and then escape immune surveillance [23]. Thus, in recent years, the PD-1–PD-L1 axis has been extensively researched in the context of cancer, because it is a promising therapeutic target. With the understanding of the immunological context of these checkpoint molecules, several monoclonal antibodies that block them have been developed (such as nivolumab, pembrolizumab, and pidilizumab, among others) and have demonstrated antitumor activity in a number of tumor types [24,25,26,27]. Unfortunately, not all types of cancer have been associated with a clinical response, emphasizing the need for a better understanding of these molecules that considers their signaling and regulation mechanisms. The upregulation of PD-L1 in cancer cells has been addressed in detail in numerous reviews [28,29,30,31]. Most of these reviews highlight genomic alterations, epigenetic modifications, oncogenic signaling, inflammatory signaling, transcriptional regulation, and post-transcriptional and post-translational modifications as mechanisms responsible for PD-L1 regulation. Recently, autophagy has been highlighted as another PD-L1 regulation mechanism [32,33,34].

**Figure 1:**
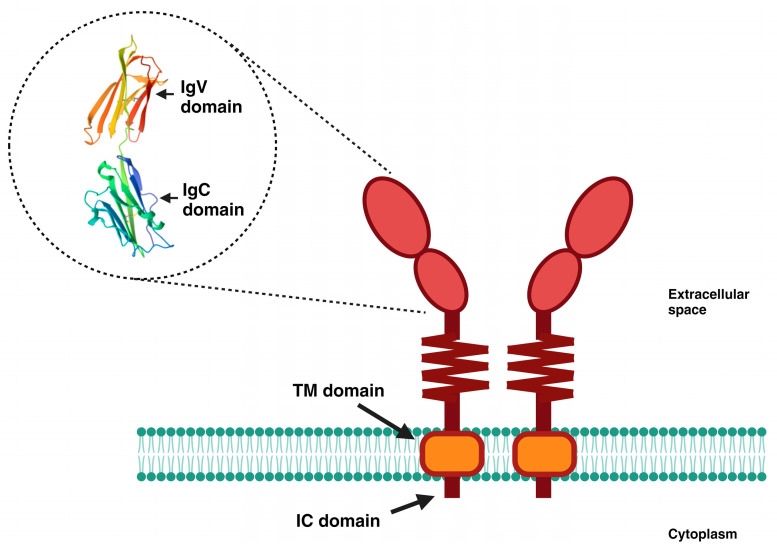
Schematic representation of PD-L1. PD-L1 is a transmembrane glycoprotein comprising 290 amino acids, grouped into two domains (IgV and IgC), followed by a short flexible stem. It has a hydrophobic transmembrane (TM) domain, as well as a small intracellular (IC) domain of 30 amino acids. The 3D image shown is from the Protein Data Bank and is available at https://www.rcsb.org/structure/3bis (accessed on 21 September 2023).

Structurally, PD-L1 is a type I transmembrane glycoprotein that belongs to the B7 family. It is a 290-amino-acid protein receptor encoded by the *CD274* gene, located on chromosome 9 in humans, and its sequence is highly conserved [35,36]. It contains IgV and IgC structure domains, a hydrophobic transmembrane domain, and a short (30 aa) cytoplasmic tail structure domain (Figure 1) that does not contain canonical signal motifs [36]. To date, the functionality of the cytoplasmic tail remains unclear. It is clear that the main studies on the PD-L1 molecule have focused on the context of its interaction with PD-1 and its inhibitory effect on the T-cell response. However, recent works have highlighted that PD-L1 can regulate tumor-intrinsic signals related to cell growth and survival, and early attempts have been made to establish the role of the intracytoplasmic domain of PD-L1 in mediating the transduction of non-canonical intracellular signals [37]. The interconnection between PD-L1 and autophagy has been documented in some reviews. Robainas and colleagues summarized and discussed the studies related to the use of immune checkpoint inhibitors in combination with autophagy inhibitors as a synergic strategy to prevent tumor progression. Cui et al. mainly highlighted the mechanisms involved in the regulation of PD-L1 via autophagy; similarly, Gao and Chen discussed the ongoing investigation into the mechanisms used by autophagy to regulate PD-L1 expression. These reviews suggest and emphasize the bidirectional regulation that exists among these biological processes and, based on this, propose new treatment strategies [34,38,39]. However, a better understanding is required concerning the non-immune intrinsic functions of PD-L1 and their potential influence on autophagy in the context of cancer. With the motivation of combatting the struggles imposed by cancer, this review is devoted to exploring and delineating new discoveries on the intrinsic immune-independent functions of PD-L1 and its impact on autophagy, and how this can influence the efficacy of current therapies or allow us to define new strategies to fight against this disease.

## 2. Overview of the Clinical Significance of PD-L1 in Cancer

Currently, the association of PD-L1 expression in various cancers with a higher risk of tumor progression is being studied, given its relationship with cancer immune escape. The PD-1–PD-L1 axis is considered to be an important control point in the treatment of these patients [40,41,42]; therefore, PD-L1 expression is one of the most important factors affecting the effectiveness of PD-1/PD-L1 blockade [43]. The main challenge is to determine whether the expression of PD-L1 and its regulation are associated with a good clinical response; currently, this question is under constant scrutiny. Numerous studies have linked PD-L1 overexpression to specific adverse clinicopathological features. Firstly, the overexpression of PD-L1 is associated with a more aggressive phenotype and a poorer prognosis in some cancers, such as gastric cancer, hepatocellular carcinoma, renal-cell carcinoma, esophageal cancer, pancreatic cancer, ovarian cancer, bladder cancer, and breast cancer [44,45,46,47,48,49,50,51]. This is due to PD-L1 generating an immunosuppressive tumor environment, avoiding T-cell activation and the subsequent antitumor immune response. Additionally, the prognostic value of PD-L1 expression in lung cancer [52,53], colorectal cancer [54,55], and melanoma [56,57] remains controversial. Several factors, such as the tumor type and stage, low immunogenicity in tumor cells, the recognition of tumor-specific antibodies as autoantigens, and tumor surface antigen modulation [58,59], could explain this discrepancy. In other types of cancer, such as thymoma, squamous-cell carcinoma of the lung, and cervical cancer, PD-L1 expression is not of prognostic value [58]. It is known that the inhibition of these checkpoints results in the apoptosis of regulatory T cells (Tregs) and enhances the immune response of effector T cells against tumor-specific antigens [60]. Nevertheless, the clinical achievements observed with PD-1/PD-L1 immune checkpoint therapy are not as expected, as patient responses to the treatment exhibit a degree of heterogeneity and unpredictability. The response to immune checkpoint inhibitors targeting PD-1/PD-L1 is influenced by complex genetic and epigenetic interactions between immune cells and tumor cells [61]. Additional, patient-specific factors, such as age and the presence of comorbidities, could influence their response to immunotherapy [40,62]. The predictive value of PD-L1 in response to PD-1/PD-L1 antibodies across various tumor types is still uncertain, and the interpretation of PD-L1 expression in tumors continues to be a subject of debate, requiring a comprehensive understanding.

## 3. PD-L1 Expression and Its Induction Factors

After the discovery of a molecule called programmed death 1 (PD-1), an important negative regulator of the immune system and a member of the immunoglobulin superfamily that contains an immunoreceptor tyrosine-based inhibitory motif (ITIM) in its cytoplasmic tail [63], an investigation was conducted to identify a B7 homolog (B7-H1), which was termed PD-1 ligand (PD-L1) [35]. Next, a second ligand for PD-1, characterized as a B7 homolog (PD-L2), was identified [64]. Together with cytotoxic T-lymphocyte-associated protein 4 (CTLA-4), PD-1 has been found to be the most reliable drug target in the treatment of advanced cancers. PD-1 is expressed on the surface of immune cells, commonly on activated T cells, B cells, and NK cells [23]. Upon ligation with PD-L1, PD-1 undergoes conformational changes that result in the recruitment of the phosphatases SHP1 and SHP2 and the dephosphorylation of downstream effectors, such as Syk, PI3K, ZAP70, and CD3ζ [65]. Moreover, the activation of PD-1 affects downstream signaling pathways such as PTEN-PI3K-Akt and RAS-MEK-ERK signaling [66,67], resulting in the inhibition of cell proliferation, cytokine production, and cytotoxic activity in effector immune cells, which promotes the immune escape of tumor cells. The expression of PD-L1 was initially detected in non-lymphoid organs, such as the heart, placenta, vascular endothelial cells, astrocytes, corneal epithelial and endothelial cells, and lungs, in both human and mouse tissues [64]. Afterwards, PD-L1 expression was identified in hematopoietic cells including T cells, B cells, macrophages, dendritic cells (DCs), and mast cells [68]. Moreover, in the tumor microenvironment, activated T cells and cancer-associated fibroblasts also express PD-L1 [59]. In fact, PD-L1 protein expression in tumor cells and in infiltrating immune cells is a biomarker in cancer immunotherapy. However, PD-L1 expression is not universally distributed across all cancer cells. Theelen et al. demonstrated the absence of PD-L1 in tumoral cells in non-small-cell lung cancer (NSCLC); they suggested that impairment of IFN-γ signaling in tumor cells could be the cause of this phenomenon [69]. The lack of PD-L1 upregulation by either tumor cells or tumor-infiltrating immune cells has also been found in biopsies from patients with other types of cancer, such as melanoma, renal-cell carcinoma, colorectal cancer, gastric cancer, and head-and-neck squamous-cell carcinoma, which display patterns with little or no tumor-infiltrating immune cell action [43]. On the other hand, the overexpression of PD-L1 can be modulated via immune mediators, oncogenic pathways, cellular stress, and epigenetic mechanisms [70]. We will now present a general overview of the main factors involved in the overexpression of the PD-L1 molecule in the context of cancer.

### 3.1. Cytokines

It is known that PD-L1 is not usually expressed in normal cells; however, its overexpression is induced in tumoral tissues [71]. This expression can be a result of the conditions of the tumor microenvironmental in relation to the adaptive immune response against cancer cells. The recruitment of immune cells in cancer tissues promotes an inflammatory microenvironment with the release of an array of inflammation mediators, including IFN-γ, that favor the expression of PD-L1 in cancer cells [72]. Interferon regulatory factor-1 (IRF-1), a transcription factor, has been associated with the positive regulation of PD-L1 [73]. Moreover, a recent study reported a synergistic effect of IFN-γ and IL-1β on PD-L1 expression in hepatocellular carcinoma, mediated by the increase in IRF-1 and the IFN-γ receptor expression induced by IL-1β [74]. In addition, the Janus kinase/signal transducer and activator of transcription (JAK/STAT1) pathways, as crucial players in promoting the cellular responses induced by IFN-γ, have been implicated in the increased expression of PD-L1 in colorectal carcinoma, gastric cancer, and pancreatic cancer [75,76,77]. Moreover, the overexpression of PD-L1 in tumor cells can be due to the activation of signal traducers induced via oncogenic pathways [78], as has been described in melanoma, with oncogenic BRAF(V600E) signaling inducing IL-1α/β production, with a subsequent upregulation of PD-L1 in the tumor stroma [79]. As the main signaling pathway involved in the regulation of PD-L1 is the JAK/STAT pathway, it is possible to hypothesize that any stimuli that trigger this pathway can cause the overexpression of PD-L1. This was proven to be true in extranodal natural killer/T-cell lymphoma, where the granulocyte–macrophage colony-stimulating factor (GM-CSF) induced a high expression of PD-L1, mediated by STAT5 mutations and JAK2 hyperphosphorylation [80]. Other molecules produced by cells engaged in the tumor microenvironment, such as CXCL5 produced by cancer-associated fibroblasts, are also responsible for increases in PD-L1 [81]. On the other hand, a recent study carried out using the tumor tissues of 148 patients with primary breast cancer correlated the high levels of PD-L1 with low circulating values of vascular endothelial growth factor (VEGF), tumor necrosis factor-beta (TNF-β), and interleukin 15 (IL-15) [82]. However, the authors highlighted that more systematic analyses are necessary because, in other types of cells, the opposite effect has been reported for IL-15. For instance, IL-2 and IL-7, in addition to IL-15, have induced the expression of PD-L1 in purified T cells in vitro [83].

### 3.2. Chemotherapy

In addition to cytokines in the tumor microenvironment inducing an increase in the expression of PD-L1, other factors have been associated with this effect. In attempts to investigate the potential effectiveness of combined chemotherapy together with anti-PD-L1 immunotherapy, some data have shown that chemotherapy has an inductor effect on PD-L1 expression. In urothelial carcinoma, the administration of neoadjuvant chemotherapy increased PD-L1 expression levels [84]. Additionally, paclitaxel treatment induced the expression of PD-L1 through the MAPK pathway in models of the human colorectal adenocarcinoma cell line SW480 and the hepatocellular carcinoma cell line HepG2 [85]. In breast cancer cells, the chemotherapeutic agent doxorubicin had a dual effect on PD-L1 expression: it was effective in downregulating the cell surface expression of PD-L1, but it also showed an upregulation of this molecule in the nucleus. This effect was associated with the involvement of the PI3K/Akt pathway [86]. Along the same line of thinking, a study on esophageal squamous-cell carcinoma reported the induction of PD-L1 after chemotherapeutic (carboplatin plus paclitaxel and 5-FU with cisplatin) treatments, mediated by the MAPK/ERK pathway [87]. 5-Fluorouracil (5-FU) also caused the induction of PD-L1 in gastrointestinal cancer cell lines [88]. Moreover, an increase in PD-L1 expression levels was demonstrated in H22 hepatoma cells treated with cisplatin. This induction was also mediated by the MAPK/ERK pathway [89].

### 3.3. Effect of the Activation of Toll-Like Receptors (TLRs) on PD-L1 Expression

Other interesting factors involved in increases in PD-L1 are still being discovered. For instance, an association between TLR activation and the PD-L1 molecule is currently being researched. Considering that, in some cancers, infections can be related to tumor regression, the impact of TLR activation on PD-L1 expression has been investigated. In neuroblastoma, TLR3 triggering has induced the upregulation of both PD-L1 and MHC class I [90]. This result is consistent with the findings in glioblastoma, in which PD-L1 upregulation is also mediated via TLR3 triggering [91]. In this context, the signaling of TLR7/8, in addition to an increase in ROS mediated by the human oncovirus EBV, initiated PD-L1 upregulation through STAT3 activation [92]. In normal CD90(+) myofibroblasts/fibroblasts (CMFs), an upregulation of PD-L1 was reported after TLR4 activation, with involvement from the MyD88/JAK2/NF-κB pathways [93]; this finding is consistent with the interplay between high PD-L1 expression and TLR4 expression levels that has been reported in colorectal cancer [94]. However, more studies are needed in order to establish the interconnection of TLR signaling and other pattern recognition receptors (PRRs) with the signaling pathways that promote PD-L1 overexpression.

### 3.4. Hypoxia

The tumor microenvironmental is highly complex, and hypoxia and metabolic factors are environmental cues to which the cells recruited in the tumor are exposed. Some in vitro studies, using models of human breast cancer cells, human prostatic carcinoma cells, mouse B16-F10 melanoma, and mouse tumor models, have revealed a relationship between hypoxia and an increase in PD-L1 levels that is dependent on the transcription factor hypoxia-inducible factor-1α (HIF-1α) [95]. This effect has also been seen in tumor-infiltrating myeloid cells, which showed an increase in PD-L1 expression under hypoxic conditions, with involvement from HIF-1α [96]. As hypoxia is the main microenvironment factor exhibited in some cancers, such as gliomas, the relationship between PD-L1 and HIF-1α has been investigated in patients. The findings provided by Ding and colleagues demonstrated that hypoxia upregulates the PD-L1 molecule by increasing HIF-1α activation [97]. These studies highlight the role of HIF-1α as a crucial player in the interplay between hypoxia and PD-L1. Accordingly, the recruitment of HIF-1α by circular RNA (circPRDM4) also increases the tumoral PD-L1 expression level in hepatocellular carcinoma [98]. Moreover, the hypoxic microenvironment has resulted in an increase in PD-L1 associated with the self-renewal of endometrial cancer stem-like cells [99]. Remarkably, in human melanoma cell lines, the expression of PD-L1 was differentially affected by hypoxia, and the role of HIF-1α was less important compared to the IFN-γ/JAK/STAT pathway stimulated by IFN-γ [100]. Interesting in vitro data, inconsistent with previous reports, indicate that, in bladder cancer, hypoxic conditions decrease the expression of PD-L1 at the RNA and protein levels, and that hypoxia abolishes the IFN-γ-induced increase in PD-L1. As this report outlined discrepancies in previous studies, the authors suggested that the upregulation of PD-L1 induced by hypoxia could be tissue-specific. Intriguingly, this effect was reverted when cells were highly confluent. The authors hypothesized that a high cellular density caused a decrease in cellular metabolism, with an impact on PD-L1 expression [101]. Previous studies reporting a decrease in the expression of surface molecules, such as TGF-beta receptors and TNF receptors, in response to an increased cell density in fibroblasts and epithelial and myeloid cell lines, could confirm the role of cellular confluency in PD-L1 expression [102,103]. Although more studies are necessary to confirm this finding, cellular density, in addition to hypoxia, could become a crucial factor in controlling the expression of PD-L1.

### 3.5. Role of Mammalian Target of Rapamycin (mTOR) in the Upregulation of PD-L1

As described above, in the tumor microenvironment, many cues promote the overexpression of PD-L1 through different signaling pathways. The mammalian target of rapamycin (mTOR) kinase is a highly conserved regulator of survival signals, and its activation in the progression of cancer has been implicated in the control of PD-L1 expression [104]. It is a serine/threonine protein kinase belonging to the phosphatidylinositol kinase-related kinase (PIKK) family [105]. mTOR is the main central governor of proliferation and cell growth and survival in mammalian cells [106]. Its role in biological processes is dependent on a collection of environmental cues, such as oxygen, stress, amino acids, and growth factors, which promote the activation of the downstream/upstream effectors associated with this molecule. The mTOR protein molecule is a core component of two protein complexes called mTORC1 (mTOR complex 1) and mTORC2 (mTOR complex 2), which elicit various downstream signaling processes to regulate cellular functions. mTORC1 is a complex composed of mTOR, RAPTOR, MLST8 (or GPL), PRAS40, Tti1/Tel2, and DEPTOR. On the other hand, MLST8, Tti1/Tel2, and DEPTOR, together with RICTOR, mSin1, and PROCTOR1/2, make up mTORC2 [107]. Both complexes respond to growth factors and participate in cell metabolism and survival; however, mTORC2 has been less extensively studied, and its mechanism of activation is not fully known. The upstream effector is PI3K, and Akt/protein kinase B (PKB) is the main downstream effector [108,109].

The stimulation of mTORC1 is dependent on the activation of the PI3K–Akt signaling pathway induced by nutrients and growth factors. Additionally, mTORC1 plays a crucial role in ribosomal synthesis, as well as the synthesis of proteins, lipids, and nucleotides. Due to the proliferation and survival functions of mTOR, it has been highly implicated in tumorigenesis [110]. In recent years, controversial evidence that highlights the role of mTOR and its downstream effectors in the regulation of PD-L1 has emerged.

The following studies have reported an upregulation of PD-L1 via the activation of mTOR in several cancer cell models: In a gastric cancer organoid model, the expression of PD-L1 induced by the hedgehog transcriptional effector GLI was mediated via the activation of the mTOR pathway [111]. In non-small-cell lung cancer (NSCLC), an increase in PD-L1 was significantly associated with the oncogenic activation of the Akt-mTOR pathway [112]. In addition, the overexpression of PD-L1 in breast cancer stem cells was dependent on the activation of mTOR by its upstream effector Notch3 [113]. Moreover, the upregulation of PD-L1 via metastasis associated with colon cancer protein 1 (MACC1) implicated the activation of the Akt/mTOR pathway in gastric cancer cells [114]. The direct interplay between PD-L1 and the activation of mTOR was also found in NSCLC cell models. Zhang and colleagues demonstrated that the pharmacological inhibition of mTOR via MTI-31/AZD8055 or rapamycin provoked a reduction in cell-surface PD-L1. With the use of specific pharmacological inhibitors, the authors established that the reduction in PD-L1 was due to proteasomal degradation induced mainly by the diminished activity of mTORC2-Akt [115]. In the same way, a close correlation between cell-intrinsic PD-L1 signals and the activation of mTOR in bladder cancer cells, promoting cell growth and metastatic cancer spread, has been demonstrated [116].

On the other hand, some findings have revealed that the upregulation of PD-L1 is closely related to the inhibition of mTOR. The inhibition of this molecule by rapamycin and Torin 1 caused an upregulation of PD-L1 in NSCLC cell lines [117]. Additionally, in a renal-cell carcinoma model, the upregulation of PD-L1 was mediated by the translocation of transcription factor EB (TFEB) induced by the inhibition of mTOR [118]. Studies using the same cancer model highlighted that the inhibition of mTOR by RADOO1, an analog of rapamycin, induced an overexpression of PD-L1 [119]. The expression of PD-L1 being dependent on the inhibition of mTOR has also been reported in non-tumor cells. Wang and colleagues showed that the inhibition of mTOR by rapamycin in endothelial cells, used as an alloimmunogenicity model, increased the expression of PD-L1 and PD-L2 molecules on the cell surface. This effect reduced the proliferation and cytokine secretion of co-cultured allogeneic memory CD4+ T cells [120]. In summary, the upregulation of PD-L1 mediated via the activation or inhibition of mTOR signaling is not fully understood, and new research is necessary in order to clarify it.

**Figure 2:**
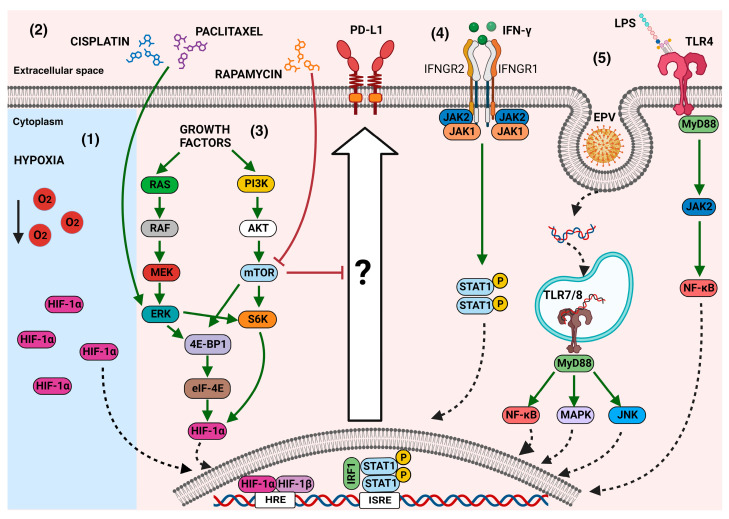
The main mechanisms that modulate PD-L1 expression in cancer cells: In the tumor microenvironment, several signals can induce PD-L1 overexpression through the activation of downstream effectors. (1) Hypoxia-inducible factor 1α (HIF-1α), which can also be induced by other stimuli, such as growth factors, has been associated with the expression of PD-L1. (2) Some chemotherapeutic agents, such as paclitaxel or cisplatin, through the phosphorylation of ERK ½, induce the expression of PD-L1; however, the complete mechanisms behind this event remain unknown. (3) The activation of mTOR through various signals, such as growth factors, triggers signaling cascades that induce the expression of PD-L1, which is decreased when the mTOR inhibitor rapamycin is used. (4) Cytokines, such as IFN-γ, induce PD-L1 expression through the JAK/STAT pathway. (5) The stimulation of some TLRs induces the activation of canonical pathways to regulate PD-L1 overexpression. Green arrows mean activation vias, red block lines mean inhibition pathways, dotted arrows mean nuclear translocation, black arrow means decreasing.

The cumulative evidence highlights the presence of multiple stimuli in the tumor microenvironment that, subsequently, could trigger an increase in PD-L1 on the cancer cell surface (Figure 2). The biological consequences of PD-L1 overexpression are under study because, recently, non-immune functions have been recognized in this molecule.

## 4. PD-L1 and Its Non-Immune Functions

The overexpression of PD-L1 in cancer cells has been largely related to the induction of the anergy and/or apoptosis of PD-1-positive T cells, through which the antitumor immunological response is subverted. However, current studies have highlighted new immune-independent functions in PD-L1 based on the signals triggered by its intracytoplasmic region. The first work related to the intrinsic immune-independent signals in PD-L1 was conducted by Azuma et al. [121]. They demonstrated that PD-L1 is implicated in cancer cells’ resistance against pro-apoptotic stimuli [121]. Moreover, the knockdown of PD-L1 expression in human gastric cancer cells significantly inhibited tumor growth and improved the cytotoxic sensitivity to CIK (cytokine-induced killer cell) therapy [122], suggesting a pivotal immune-independent role of PD-L1. Later, three well-conserved sequence motifs (“RMLDVEKC”, “DTSSK”, and “QFEET”) were identified in the intracytoplasmic region of PD-L1 associated with its non-immune functions. The RMLDVEKC and DTSSK sequences were involved in cancer cells’ resistance to apoptotic effects induced by interferons [37]. The regulation of the biological behavior of cancer cells by PD-L1 also covers the induction of the epithelial–mesenchymal transition, which has been reported in lung adenocarcinoma, breast cancer cells, and thyroid cancer, among others [123,124,125]. Moreover, resistance to radiotherapy and chemotherapy has been associated with PD-L1 expression in non-small-cell lung cancer through the regulation of the DNA damage response [126,127] and, importantly, the direct regulation of tumor cell metabolism by PD-L1 has been described in sarcoma tumors [128]. Although it is clear that PD-L1 has the function of governing the intracellular signals involved in the survival and proliferation of cancer cells independent of T cells, the mechanism for this has not, to date, been well defined.

Currently, there is little information regarding the downstream signaling effectors of PD-L1, and it is unknown how this molecule activates or deactivates signals that culminate in the survival of cancer cells. The increased detection of Akt-1 in co-immunoprecipitation with PD-L1 in HNSCC cell lines, along with the recent description of the intracellular signalosome of PD-L1, highlight how it functions immune-independently, regulated by the mTOR-Akt pathway [37,129], which could explain the impact of the intrinsic functions of PD-L1 on the cellular processes that culminate in the sustained proliferation and growth of cancer cells.

The intrinsic effect of PD-L1 on tumor growth has been described in various cancer types. In melanoma and in ovarian cancer cells, PD-L1 stimulates tumor cell proliferation and alters autophagy through the regulation of mTOR [130], in addition to promoting the generation of tumor-initiating cells driven by mTORC1 [131]. In leukemia, the downregulated PD-L1 expression inhibits cell proliferation with the induction of G2/M-phase arrest and apoptosis through the PI3K-Akt signaling pathway [132]. The involvement of the Akt-mTOR pathway as a downstream effector triggered by PD-L1 has also been reported in processes related to invasion and metastasis, such as the epithelial–mesenchymal transition (EMT) process in hypopharyngeal squamous-cell carcinoma (HSCC) [133], as well as in chemotherapy resistance in bladder cancers [116].

In addition to the modulation of signals triggered via mTOR activation, new discoveries have been described. Experiments conducted on cancer cell lines have demonstrated that the conserved RMLDVEKC motif of the PD-L1 intracytoplasmic tail is related to the inhibition of STAT3 Y705 phosphorylation and the prevention of STAT3 upregulation in the line to avoid IFN cytotoxicity [134]. The investigation of signal transduction events induced by PD-L1 is ongoing, and further studies are needed in order to discover the target cytoplasmic effectors triggered by the activation of the PD-L1 molecule, as well as how those effectors are interconnected to modulate the biological events implicated in cancer progression.

## 5. Autophagy Is Controlled by Intrinsic Signaling of PD-L1

**Figure 3:**
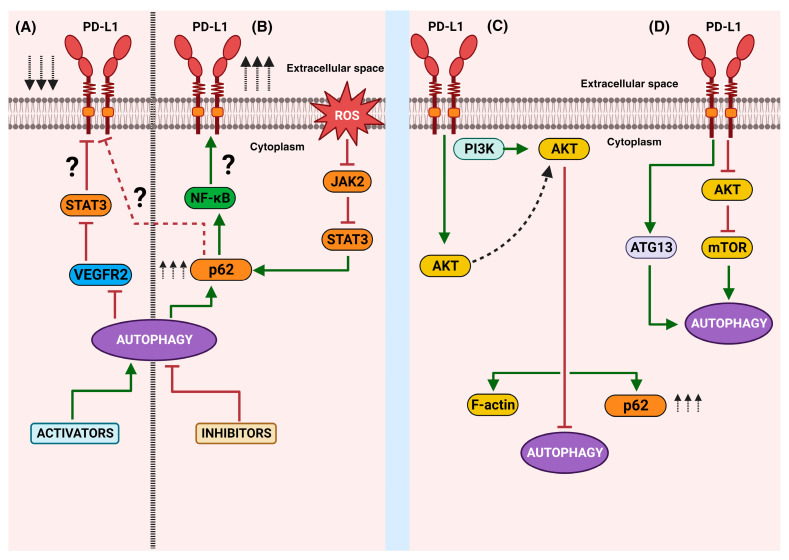
A schematic representation of the bidirectional autophagy/PD-L1 relationship: The **right** side shows the main findings related to the effects of the activation (**A**) or inhibition (**B**) of autophagy on PD-L1 expression. The **left** side represents the effects of the intrinsic non-immune functions of PD-L1 and the known downstream molecules that culminate in the disruption (**C**) or activation (**D**) of autophagy. Green arrows mean activation vias, red block lines mean inhibition pathways, red dotted line means hypothetical pathway, black arrows aiming up mean increasing, black arrows aiming down mean decreasing.

Remarkably, an association between autophagy and PD-L1 expression has recently emerged. Despite there being few reports on this topic, a bidirectional effect along the autophagy–PD-L1 axis seems possible. However, the approach that explores the role of autophagy as a regulatory pathway of PD-L1 expression is the most known. Indeed, a study found that pharmacological inhibition of autophagy increases the expression of PD-L1 in gastric cancer through the p62/SQSTM1-NF-κB pathway [32]. In line with this result, a recent work reported that andrographolide, a principal active component in *Andrographis paniculate*, caused the selective autophagic degradation of PD-L1, mediated by the inhibition of STAT3 phosphorylation and the accumulation of p62 [135]. In addition, verteporfin suppressed the intrinsically expressed PD-L1 and the interferon-induced PD-L1 expression though autophagy [33]. Moreover, the autophagy induced by apatinib decreased PD-L1 expression in lung cancer cells through the ROS/Nrf2/p62 pathway [136]. The PD-L1 molecule and its relationship with ATG proteins have also started to be explored in relation to tumor cell invasion. Interesting data indicate that the overexpression of ATG7 elevates PD-L1 protein expression through the downregulation of forkhead box (FOX)O3a protein expression and a decrease in miR-145 promoter transcription [137]. Moreover, the invasion and metastasis associated with tumor-derived small extracellular vesicle (sEV) PD-L1 could be avoided because autophagy activation via temsirolimus (TEM), an FDA-approved anticancer drug, suppressed the cellular PD-L1 levels and PD-L1 levels in sEVs in a dose-dependent manner in cellular models of breast cancer [138]. Together, these studies strongly suggest that the activation of autophagy regulates PD-L1 expression (Figure 3A,B). It is known that a high expression of PD-L1 is related to poor prognoses and low survival rates in patients. Therefore, several ongoing studies propose the modulation of autophagy, in combination with immunotherapy, as an alternative strategy for treating cancer in its advanced stages.

The other approach to the autophagy–PD-L1 interplay is to explore the effect of the intracellular signaling of PD-L1 on autophagy. The activation or inhibition of autophagy is a process closely related to both the mTORC1 and mTORC2 complexes. mTORC2 indirectly inhibits autophagy through the Akt1–FOXO3a axis, while mTORC1 inhibits autophagy through the direct hyperphosphorylation of ULK1 and ATG13 under nutrient-rich conditions [139]. As recent reports have demonstrated that mTOR is a potential target for the signaling of the intracytoplasmic tail of PD-L1, it has been suggested that autophagy could be indirectly controlled by the non-immune functions of PD-L1. It is well known that autophagy is negatively regulated by mTOR activation. This fact is consistent with the findings in mouse melanoma and in both mouse and human ovarian cancer cells, in which a high expression of PD-L1 was correlated with high mTORC1 activity and reduced basal autophagy [130,140]. However, the intrinsic effect of PD-L1 on autophagy seems to depend on the type of cell. In bladder cells, autophagy is promoted by intrinsic PD-L1 signals consistent with the dampened mTORC1 response and changes in the LC3-II/LC3-I ratio. The use of chloroquine and 3-methyladenine as pharmacologic autophagy inhibitors confirmed that both steady-state autophagy and autophagic flux are promoted by bladder-cell-intrinsic PD-L1 [116], contrary to the results described in melanoma and ovarian cancer cells, where starvation-induced autophagy was inhibited by an increase in mTORC1 and mTORC2 signals [130]. Furthermore, the increase in PD-L1 overexpression via neoadjuvant chemotherapy (NACT) in gastric cancer induced a decrease in the Akt/mTOR signaling cascade, which was associated with the initiation of autophagy [141]. It is known that autophagy is a complex process, involving a series of molecules that activate in cascade; unfortunately, a direct relationship between intrinsic PD-L1 signaling and a specific autophagy protein remains unknown in a lot of cancers, but in some cancers this research is in progress.

The dissection of target molecules downstream of PD-L1 signaling has been advanced in PD-L1-overexpressed U251 glioma cells, in which expression was positively correlated with the PI3K/Akt pathway, p62/SQSTM1, and β-actin. To elucidate the molecular mechanism of PD-L1′s action, a PD-L1 series truncated via deletion was used. The results showed that a PD-L1 128-237 fragment was required for preferential binding of Akt1 and Akt2. The PD-L1–Akt interaction led to a modification in the cellular morphology depending on the F-actin cytoskeleton and, importantly, it showed lower Beclin-1 and LC3 levels, consistent with the suppression of autophagy and an increase in p62 [142]. These studies are controversial, and further experimentation is required in order to attain a general view of the role of PD-L1 in autophagy. These contradictory results could be due to the type of cancer, the stage of the cancer, or the downstream molecules activated after PD-L1 signaling. Furthermore, it is necessary to establish and clarify the interconnection between PD-L1 and both mTOR complexes, as well as how these molecules influence autophagy.

The control of autophagy via the intrinsic effect of PD-L1 is dependent on the activation of positive or negative regulatory molecules. A relevant study using hepatocellular carcinoma models correlated tumor growth with the induction of autophagy. Later immune precipitation assays showed an interaction of PD-L1 with ATG13 (an accessory protein of the ULK1 kinase complex, an initiator of autophagy), which caused an increase in autophagy [143]. These results indicate that the intrinsic signaling of PD-L1 can culminate in the activation of key molecules for triggering autophagy (Figure 3C,D), thus allowing cells to tolerate unfavorable conditions and continue to grow and escape the immune response.

Together, these studies highlight the bidirectional interplay between PD-L1 and autophagy (Figure 3). Currently, few molecular players and signaling pathways have been recognized from both angles. Mechanistically, the activation or inhibition of autophagy can, in turn, regulate the expression of PD-L1 through the involvement of STAT3, NF-κB, or p62. On the other hand, the intrinsic functions of PD-L1′s downstream signaling can disrupt or activate autophagy, with the participation of mTOR, Akt, p62, or ATG13.

To date, the research on the signaling of PD-L1 that culminates in the activation or deactivation of autophagy is in its infancy. The few studies are controversial, and further research is necessary in order to obtain a clear view of the effect of PD-L1 signaling on autophagy, as well as the molecules involved. In advanced stages of cancer, the negative regulation of autophagy via high levels of PD-L1 could be an advantageous intervention for disrupting cancer cells’ sources of nutrients and energy, as well as their adaptability to stress. However, the clinical efficiency should be evaluated considering the participation of mTOR and other crucial players whose perpetual activation could control cell growth and cancer cell metabolism. Furthermore, the inhibition of autophagy also blocks the normal catabolic degradation of PD-L1 and could inhibit the functionality of immune cells. Due to the complex dual role of the autophagy–PD-L1 axis, potential clinical interventions must tread a delicate balance regarding the activation or inhibition of the processes involved, as well as the stage of the tumor and the types of cells affected by the treatments, in order to be successful.

## 6. Conclusions

There have been scientific and valid considerations related to the relevance of inducing the upregulation of PD-L1 in tumor cells, mainly because a better recognition by the therapeutic anti-PD-L1 could be achieved. However, the new research that highlights the role of the non-immune intrinsic functions of PD-L1 in the growth and survival of cancer cells forces us to reconsider this hypothesis. Moreover, the low response rates (less than 40%) of patients to PD-1/PD-L1 blockade therapy illustrate the need for a better understanding of the functions of PD-L1 and the biological impact of its negative or positive regulation in the tumor environment. The presence of numerous cues in the tumor microenvironment that induce the overexpression of PD-L1 highlights the potential increase in intracellular signals triggered by this molecule. What biological phenomena are initiated after the signaling of PD-L1? Some of them, such as apoptosis, chemotherapy resistance, and tumoral metabolism, are being studied, with controversial results in some cases. The latest research on this topic has indicated that autophagy is another potential target process for PD-L1 signaling. Although much remains to be elucidated, PD-L1 seems to activate more than one signaling pathway and, therefore, can turn autophagy on or off. In some types of cancer, such as glioblastoma, renal-cell carcinoma, lung cancer, colon adenocarcinoma, and hepatocellular carcinoma, among others, PD-L1 has been associated with a poor prognosis. On the other hand, increased autophagy often supports tumor cells’ survival and growth. Thus, the redundancy of these processes could be a vicious circle that must be pharmacologically broken. In fact, there are ongoing clinical trials for NSCLC, breast cancer, metastatic pancreatic adenocarcinoma, advanced melanoma, pancreatic cancer, and metastatic renal-cell carcinoma with a combination of PD-L1 and autophagy inhibitors. However, the relevance of pharmacologically modulating the expression of PD-L1 should be evaluated, with a complete view of its implications for autophagy. This review emphasizes our current understanding related to the regulation of autophagy via the intrinsic functions of overexpressed PD-L1, but whether or not the downregulation of PD-L1 controls autophagy remains unknown. In addition, further experiments are needed in order to elucidate the molecules involved in the downstream signaling of PD-L1 that could culminate in the activation or deactivation of the autophagy machinery. Meanwhile, these findings can help in the design of new combined therapy strategies to improve the efficacy of cancer treatments.

## 7. Methodology

This review was written using information sourced from PubMed. The keywords searched were “autophagy AND PD-L1”, “autophagy AND cancer”, “PD-L1 AND mTOR”, and “PD-L1 AND hypoxia”. The information was filtered to select works published in the last 10 years, except for some references related to general information about autophagy, PD-L1, mTOR, and cancer.

## Data Availability

The data presented in this study are available in PubMed.

## References

[1] Xiao Y., Cai W., Le W. (2020). Autophagy and Bacterial Infection. Autophagy: Biology and Diseases: Clinical Science.

[2] Park H., Kang J.H., Lee S. (2020). Autophagy in Neurodegenerative Diseases: A Hunter for Aggregates. Int. J. Mol. Sci..

[3] Deretic V. (2021). Autophagy in Inflammation, Infection, and Immunometabolism. Immunity.

[4] Levine B., Kroemer G. (2019). Biological Functions of Autophagy Genes: A Disease Perspective. Cell.

[5] Li X., He S., Ma B. (2020). Autophagy and Autophagy-Related Proteins in Cancer. Mol. Cancer.

[6] Poillet-Perez L., White E. (2019). Role of Tumor and Host Autophagy in Cancer Metabolism. Genes Dev..

[7] Marsh T., Kenific C.M., Suresh D., Gonzalez H., Shamir E.R., Mei W., Tankka A., Leidal A.M., Kalavacherla S., Woo K. (2020). Autophagic Degradation of NBR1 Restricts Metastatic Outgrowth during Mammary Tumor Progression. Dev. Cell.

[8] Wang Y., Xie J., Wang H., Huang H., Xie P. (2017). Beclin-1 Suppresses Gastric Cancer Progression by Promoting Apoptosis and Reducing Cell Migration. Oncol. Lett..

[9] Yue Z., Jin S., Yang C., Levine A.J., Heintz N. (2003). Beclin 1, an Autophagy Gene Essential for Early Embryonic Development, Is a Haploinsufficient Tumor Suppressor. Proc. Natl. Acad. Sci. USA.

[10] Wijshake T., Zou Z., Chen B., Zhong L., Xiao G., Xie Y., Doench J.G., Bennett L., Levine B. (2021). Tumor-Suppressor Function of Beclin 1 in Breast Cancer Cells Requires E-Cadherin. Proc. Natl. Acad. Sci. USA.

[11] Ohsumi Y. (2014). Historical landmarks of autophagy research. Cell Res..

[12] Klionsky D.J., Cregg J.M., Dunn W.A., Emr S.D., Sakai Y., Sandoval I.V., Sibirny A., Subramani S., Thumm M., Veenhuis M. (2003). A unified nomenclature for yeast autophagy-related genes. Dev. Cell.

[13] Takamura A., Komatsu M., Hara T., Sakamoto A., Kishi C., Waguri S., Eishi Y., Hino O., Tanaka K., Mizushima N. (2011). Autophagy-Deficient Mice Develop Multiple Liver Tumors. Genes Dev..

[14] Lum J.J., Bauer D.E., Kong M., Harris M.H., Li C., Lindsten T., Thompson C.B. (2005). Growth factor regulation of autophagy and cell survival in the absence of apoptosis. Cell.

[15] Jin S., DiPaola R.S., Mathew R., White E. (2007). Metabolic catastrophe as a means to cancer cell death. J. Cell Sci..

[16] Degenhardt K., Mathew R., Beaudoin B., Bray K., Anderson D., Chen G., Mukherjee C., Shi Y., Gélinas C., Fan Y. (2006). Autophagy promotes tumor cell survival and restricts necrosis, inflammation, and tumorigenesis. Cancer Cell.

[17] Huo Y., Cai H., Teplova I., Bowman-Colin C., Chen G., Price S., Barnard N., Ganesan S., Karantza V., White E. (2013). Autophagy opposes p53-mediated tumor barrier to facilitate tumorigenesis in a model of PALB2-associated hereditary breast cancer. Cancer Discov..

[18] Guo J.Y., Xia B., White E. (2013). Autophagy-Mediated Tumor Promotion. Cell.

[19] White E., Lattime E.C., Guo J.Y. (2021). Autophagy Regulates Stress Responses, Metabolism, and Anticancer Immunity. Trends Cancer.

[20] Yamamoto K., Venida A., Yano J., Biancur D.E., Kakiuchi M., Gupta S., Sohn A.S.W., Mukhopadhyay S., Lin E.Y., Parker S.J. (2020). Autophagy Promotes Immune Evasion of Pancreatic Cancer by Degrading MHC-I. Nature.

[21] Barnard R.A., Regan D.P., Hansen R.J., Maycotte P., Thorburn A., Gustafson D.L. (2016). Autophagy Inhibition Delays Early but Not Late-Stage Metastatic Disease. J. Pharmacol. Exp. Ther..

[22] Pardoll D.M. (2012). The Blockade of Immune Checkpoints in Cancer Immunotherapy. Nat. Rev. Cancer.

[23] Ai L., Xu A., Xu J., Xu J. (2020). Roles of PD-1/PD-L1 Pathway: Signaling, Cancer, and Beyond. Regulation of Cancer Immune Checkpoints: Molecular and Cellular Mechanisms and Therapy.

[24] Kiasari B.A., Abbasi A., Ghasemi Darestani N., Adabi N., Moradian A., Yazdani Y., Sadat Hosseini G., Gholami N., Janati S. (2022). Combination Therapy with Nivolumab (Anti-PD-1 Monoclonal Antibody): A New Era in Tumor Immunotherapy. Int. Immunopharmacol..

[25] Kwok G., Yau T.C.C., Chiu J.W., Tse E., Kwong Y.-L. (2016). Pembrolizumab (Keytruda). Hum. Vaccin. Immunother..

[26] Westin J.R., Chu F., Zhang M., Fayad L.E., Kwak L.W., Fowler N., Romaguera J., Hagemeister F., Fanale M., Samaniego F. (2014). Safety and Activity of PD1 Blockade by Pidilizumab in Combination with Rituximab in Patients with Relapsed Follicular Lymphoma: A Single Group, Open-Label, Phase 2 Trial. Lancet Oncol..

[27] Jiang Y., Chen M., Nie H., Yuan Y. (2019). PD-1 and PD-L1 in Cancer Immunotherapy: Clinical Implications and Future Considerations. Hum. Vaccin. Immunother..

[28] Chen J., Jiang C.C., Jin L., Zhang X.D. (2016). Regulation of PD-L1: A Novel Role of pro-Survival Signaling in Cancer. Ann. Oncol..

[29] Yi M., Niu M., Xu L., Luo S., Wu K. (2021). Regulation of PD-L1 Expression in the Tumor Microenvironment. J. Hematol. Oncol..

[30] Antonangeli F., Natalini A., Garassino M.C., Sica A., Santoni A., Di Rosa F. (2020). Regulation of PD-L1 Expression by NF-ΚB in Cancer. Front. Immunol..

[31] Bailly C. (2020). Regulation of PD-L1 Expression on Cancer Cells with ROS-Modulating Drugs. Life Sci..

[32] Wang X., Wu W.K.K., Gao J., Li Z., Dong B., Lin X., Li Y., Li Y., Gong J., Qi C. (2019). Autophagy Inhibition Enhances PD-L1 Expression in Gastric Cancer. J. Exp. Clin. Cancer Res..

[33] Liang J., Wang L., Wang C., Shen J., Su B., Marisetty A.L., Fang D., Kassab C., Jeong K.J., Zhao W. (2020). Verteporfin Inhibits PD-L1 through Autophagy and the STAT1–IRF1–TRIM28 Signaling Axis, Exerting Antitumor Efficacy. Cancer Immunol. Res..

[34] Cui Y., Shi J., Cui Y., Zhu Z., Zhu W. (2023). The Relationship between Autophagy and PD-L1 and Their Role in Antitumor Therapy. Front. Immunol..

[35] Freeman G.J., Long A.J., Iwai Y., Bourque K., Chernova T., Nishimura H., Fitz L.J., Malenkovich N., Okazaki T., Byrne M.C. (2000). Engagement of the Pd-1 Immunoinhibitory Receptor by a Novel B7 Family Member Leads to Negative Regulation of Lymphocyte Activation. J. Exp. Med..

[36] Keir M.E., Butte M.J., Freeman G.J., Sharpe A.H. (2008). PD-1 and Its Ligands in Tolerance and Immunity. Annu. Rev. Immunol..

[37] Escors D., Gato-Cañas M., Zuazo M., Arasanz H., García-Granda M.J., Vera R., Kochan G. (2018). The Intracellular Signalosome of PD-L1 in Cancer Cells. Signal Transduct. Target. Ther..

[38] Robainas M., Otano R., Bueno S., Ait-Oudhia S. (2017). Understanding the Role of PD-L1/PD1 Pathway Blockade and Autophagy in Cancer Therapy. OncoTargets Ther..

[39] Gao L., Chen Y. (2021). Autophagy Controls Programmed Death-Ligand 1 Expression on Cancer Cells. Biomed. Rep..

[40] Brahmer J.R., Tykodi S.S., Chow L.Q., Hwu W.J., Topalian S.L., Hwu P., Drake C.G., Camacho L.H., Kauh J., Odunsi K. (2012). Safety and activity of anti-PD-L1 antibody in patients with advanced cancer. N. Engl. J. Med..

[41] Kim D.H., Kim H., Choi Y.J., Kim S.Y., Lee J.E., Sung K.J., Sung Y.H., Pack C.G., Jung M.K., Han B. (2019). Exosomal PD-L1 promotes tumor growth through immune escape in non-small cell lung cancer. Exp. Mol. Med..

[42] Zhou Q.H., Li K.W., Chen X., He H.X., Peng S.M., Peng S.R., Wang Q., Li Z.A., Tao Y.R., Cai W.L. (2020). HHLA2 and PD-L1 co-expression predicts poor prognosis in patients with clear cell renal cell carcinoma. J. Immunother. Cancer.

[43] Herbst R.S., Soria J.C., Kowanetz M., Fine G.D., Hamid O., Gordon M.S., Sosman J.A., McDermott D.F., Powderly J.D., Gettinger S.N. (2014). Predictive correlates of response to the anti-PD-L1 antibody MPDL3280A in cancer patients. Nature.

[44] Hou J., Yu Z., Xiang R., Li C., Wang L., Chen S., Li Q., Chen M., Wang L. (2014). Correlation between infiltration of FOXP3+ regulatory T cells and ex-pression of B7-H1 in the tumor tissues of gastric cancer. Exp. Mol. Pathol..

[45] Jung H.I., Jeong D., Ji S., Ahn T.S., Bae S.H., Chin S., Chung J.C., Kim H.C., Lee M.S., Baek M.J. (2017). Overexpression of PD-L1 and PD-L2 Is Associated with Poor Prognosis in Patients with Hepatocellular Carcinoma. Cancer Res. Treat..

[46] Carlsson J., Sundqvist P., Kosuta V., Fält A., Giunchi F., Fiorentino M., Davidsson S. (2020). PD-L1 Expression is Associated with Poor Prognosis in Renal Cell Carcinoma. Appl. Immunohistochem. Mol. Morphol..

[47] Zhang F., Zhu X., Zhang Q., Zhou P., Hao L. (2021). Programmed cell death-ligand 1 expression predicts poor treatment response and prognostic value in esophageal squamous cell car-cinoma patients without esophagectomy. Aging.

[48] Nomi T., Sho M., Akahori T., Hamada K., Kubo A., Kanehiro H., Nakamura S., Enomoto K., Yagita H., Azuma M. (2007). Clinical significance and therapeutic potential of the programmed death-1 ligand/programmed death-1 pathway in human pancreatic cancer. Clin. Cancer Res..

[49] Hamanishi J., Mandai M., Iwasaki M., Okazaki T., Tanaka Y., Yamaguchi K., Higuchi T., Yagi H., Takakura K., Minato N. (2007). Programmed cell death 1 ligand 1 and tumor-infiltrating CD8^+^ T lymphocytes are prognostic factors of human ovarian cancer. Proc. Natl. Acad. Sci. USA.

[50] Nakanishi J., Wada Y., Matsumoto K., Azuma M., Kikuchi K., Ueda S. (2007). Overexpression of B7-H1 (PD-L1) significantly associates with tumor grade and postoperative prognosis in human urothelial cancers. Cancer Immunol. Immunother..

[51] Zhang M., Sun H., Zhao S., Wang Y., Pu H., Wang Y., Zhang Q. (2017). Expression of PD-L1 and prognosis in breast cancer: A meta-analysis. Oncotarget.

[52] Mu C.Y., Huang J.A., Chen Y., Chen C., Zhang X.G. (2011). High expression of PD-L1 in lung cancer may contribute to poor prognosis and tumor cells immune escape through suppressing tumor infiltrating dendritic cells maturation. Med. Oncol..

[53] Velcheti V., Schalper K.A., Carvajal D.E., Anagnostou V.K., Syrigos K.N., Sznol M., Herbst R.S., Gettinger S.N., Chen L., Rimm D.L. (2014). Programmed death ligand-1 expression in non-small cell lung cancer. Lab. Investig..

[54] Shi S.J., Wang L.J., Wang G.D., Guo Z.Y., Wei M., Meng Y.L., Yang A.G., Wen W.H. (2013). B7-H1 expression is associated with poor prognosis in colorectal carcinoma and regulates the proliferation and invasion of HCT116 colorectal cancer cells. PLoS ONE.

[55] Droeser R.A., Hirt C., Viehl C.T., Frey D.M., Nebiker C., Huber X., Zlobec I., Eppenberger-Castori S., Tzankov A., Rosso R. (2013). Clinical impact of programmed cell death ligand 1 expression in colorectal cancer. Eur. J. Cancer.

[56] Hino R., Kabashima K., Kato Y., Yagi H., Nakamura M., Honjo T., Okazaki T., Tokura Y. (2010). Tumor cell expression of programmed cell death-1 ligand 1 is a prognostic factor for malignant melanoma. Cancer.

[57] Taube J.M., Anders R.A., Young G.D., Xu H., Sharma R., McMiller T.L., Chen S., Klein A.P., Pardoll D.M., Topalian S.L. (2012). Colocalization of inflammatory response with B7-h1 expression in human melanocytic lesions supports an adaptive resistance mechanism of immune escape. Sci. Transl. Med..

[58] Wang X., Teng F., Kong L., Yu J. (2016). PD-L1 expression in human cancers and its association with clinical outcomes. OncoTargets Ther..

[59] Jiang X., Wang J., Deng X., Xiong F., Ge J., Xiang B., Wu X., Ma J., Zhou M., Li X. (2019). Role of the Tumor Microenvironment in PD-L1/PD-1-Mediated Tumor Immune Escape. Mol. Cancer.

[60] Mucileanu A., Chira R., Mircea P.A. (2021). PD-1/PD-L1 expression in pancreatic cancer and its implication in novel therapies. Med. Pharm. Rep..

[61] Moise J., Murthy J., Dabir D., Yu S., Kisto F., Herron E., Aulakh S. (2022). Mechanisms of Resistance and Strategies to Combat Resistance in PD-(L)1 Blockade. Immuno.

[62] Zeng X., Zhu S., Xu C., Wang Z., Su X., Zeng D., Long H., Zhu B. (2020). Effect of Comorbidity on Outcomes of Patients with Advanced Non-Small Cell Lung Cancer Undergoing Anti-PD1 Immunotherapy. Med. Sci. Monit..

[63] Ishida Y., Agata Y., Shibahara K., Honjo T. (1992). Induced Expression of PD-1, a Novel Member of the Immunoglobulin Gene Superfamily, upon Programmed Cell Death. EMBO J..

[64] Latchman Y., Wood C.R., Chernova T., Chaudhary D., Borde M., Chernova I., Iwai Y., Long A.J., Brown J.A., Nunes R. (2001). PD-L2 Is a Second Ligand for PD-1 and Inhibits T Cell Activation. Nat. Immunol..

[65] Rotte A. (2019). Combination of CTLA-4 and PD-1 blockers for treatment of cancer. J. Exp. Clin. Cancer Res..

[66] Parry R.V., Chemnitz J.M., Frauwirth K.A., Lanfranco A.R., Braunstein I., Kobayashi S.V., Linsley P.S., Thompson C.B., Riley J.L. (2005). CTLA-4 and PD-1 receptors inhibit T-cell activation by distinct mechanisms. Mol. Cell. Biol..

[67] Patsoukis N., Brown J., Petkova V., Liu F., Li L., Boussiotis V.A. (2012). Selective effects of PD-1 on Akt and Ras pathways regulate molecular components of the cell cycle and inhibit T cell proliferation. Sci. Signal..

[68] Sun C., Mezzadra R., Schumacher T.N. (2018). Regulation and Function of the PD-L1 Checkpoint. Immunity.

[69] Theelen W.S.M.E., Kuilman T., Schulze K., Zou W., Krijgsman O., Peters D.D.G.C., Cornelissen S., Monk-horst K., Sarma P., Sumiyoshi T. (2019). Absence of PD-L1 expression on tumor cells in the context of an activated immune infiltrate may indicate impaired IFNγ signaling in non-small cell lung cancer. PLoS ONE.

[70] Zhang H., Dai Z., Wu W., Wang Z., Zhang N., Zhang L., Zeng W.-J., Liu Z., Cheng Q. (2021). Regulatory Mechanisms of Immune Checkpoints PD-L1 and CTLA-4 in Cancer. J. Exp. Clin. Cancer Res..

[71] Dong H., Strome S.E., Salomao D.R., Tamura H., Hirano F., Flies D.B., Roche P.C., Lu J., Zhu G., Tamada K. (2002). Tumor-Associated B7-H1 Promotes T-Cell Apoptosis: A Potential Mechanism of Immune Evasion. Nat. Med..

[72] Spranger S., Spaapen R.M., Zha Y., Williams J., Meng Y., Ha T.T., Gajewski T.F. (2013). Up-Regulation of PD-L1, IDO, and Tregs in the Melanoma Tumor Microenvironment Is Driven by CD8^+^ T Cells. Sci. Transl. Med..

[73] Lee S.J., Jang B.C., Lee S.W., Yang Y.I., Suh S.I., Park Y.M., Oh S., Shin J.G., Yao S., Chen L. (2006). Interferon Regulatory Factor-1 Is Prerequisite to the Constitutive Expression and IFN-γ-Induced Upregulation of B7-H1 (CD274). FEBS Lett..

[74] Numata Y., Akutsu N., Ishigami K., Koide H., Wagatsuma K., Motoya M., Sasaki S., Nakase H. (2022). Synergistic Effect of IFN-γ and IL-1β on PD-L1 Expression in Hepatocellular Carcinoma. Biochem. Biophys. Rep..

[75] Mimura K., The J.L., Okayama H., Shiraishi K., Kua L.F., Koh V., Smoot D.T., Ashktorab H., Oike T., Suzuki Y. (2018). PD-L1 Expression Is Mainly Regulated by Interferon Gamma Associated with JAK-STAT Pathway in Gastric Cancer. Cancer Sci..

[76] Imai D., Yoshizumi T., Okano S., Itoh S., Ikegami T., Harada N., Aishima S., Oda Y., Maehara Y. (2019). IFN-γ Promotes Epithelial-Mesenchymal Transition and the Expression of PD-L1 in Pancreatic Cancer. J. Surg. Res..

[77] Zhao T., Li Y., Zhang J., Zhang B. (2020). PD/L1 Expression Increased by IFN-γ via JAK2/STAT1 Signaling and Predicts a Poor Survival in Colorectal Cancer. Oncol. Lett..

[78] Ribas A. (2015). Adaptive Immune Resistance: How Cancer Protects from Immune Attack. Cancer Discov..

[79] Khalili J.S., Liu S., Rodríguez-Cruz T.G., Whittington M., Wardell S., Liu C., Zhang M., Cooper Z.A., Frederick D.T., Li Y. (2012). Oncogenic BRAF(V600E) promotes stromal cell-mediated immunosuppression via induction of interleukin-1 in melanoma. Clin. Cancer Res..

[80] Rong Q., Wang F., Guo Z., Hu Y., An S., Luo M., Zhang H., Wu S., Huang H., Fu L. (2021). GM-CSF Mediates Immune Evasion via Upregulation of PD-L1 Expression in Extranodal Natural Killer/T Cell Lymphoma. Mol. Cancer.

[81] Li Z., Zhou J., Zhang J., Li S., Wang H., Du J. (2019). Cancer-Associated Fibroblasts Promote PD-L1 Expression in Mice Cancer Cells via Secreting CXCL5. Int. J. Cancer.

[82] Cierna Z., Smolkova B., Cholujova D., Gronesova P., Miklikova S., Cihova M., Plava J., Mego M. (2021). Decreased Levels of Circulating Cytokines VEGF, TNF-β and IL-15 Indicate PD-L1 Overexpression in Tumours of Primary Breast Cancer Patients. Sci. Rep..

[83] Kinter A.L., Godbout E.J., McNally J.P., Sereti I., Roby G.A., O’Shea M.A., Fauci A.S. (2008). The common gamma-chain cytokines IL-2, IL-7, IL-15, and IL-21 induce the ex-pression of programmed death-1 and its ligands. J. Immunol..

[84] McDaniel A.S., Alva A., Zhan T., Xiao H., Cao X., Gursky A., Siddiqui J., Chinnaiyan A.M., Jiang H., Lee C.T. (2016). Expression of PDL1 (B7-H1) Before and After Neoadjuvant Chemotherapy in Urothelial Carcinoma. Eur. Urol. Focus.

[85] Gong W., Song Q., Lu X., Gong W., Zhao J., Min P., Yi X. (2011). Paclitaxel Induced B7-H1 Expression in Cancer Cells via the MAPK Pathway. J. Chemother..

[86] Ghebeh H., Lehe C., Barhoush E., Al-Romaih K., Tulbah A., Al-Alwan M., Hendrayani S.F., Manogaran P., Alaiya A., Al-Tweigeri T. (2010). Doxorubicin Downregulates Cell Surface B7-H1 Expression and Upregulates Its Nuclear Expression in Breast Cancer Cells: Role of B7-H1 as an Anti-Apoptotic Molecule. Breast Cancer Res..

[87] Ng H.Y., Li J., Tao L., Lam A.K., Chan K.W., Ko J.M.Y., Yu V.Z., Wong M., Li B., Lung M.L. (2018). Chemotherapeutic Treatments Increase PD-L1 Expression in Esophageal Squamous Cell Carcinoma through EGFR/ERK Activation. Transl. Oncol..

[88] Van Der Kraak L., Goel G., Ramanan K., Kaltenmeier C., Zhang L., Normolle D.P., Freeman G.J., Tang D., Nason K.S., Davison J.M. (2016). 5-Fluorouracil Upregulates Cell Surface B7-H1 (PD-L1) Expression in Gastrointestinal Cancers. J. Immunother. Cancer.

[89] Qin X., Liu C., Zhou Y., Wang G. (2010). Cisplatin Induces Programmed Death-1-Ligand 1(PD-L1) over-Expression in Hepatoma H22 Cells via Erk /MAPK Signaling Pathway. Cell. Mol. Biol..

[90] Boes M., Meyer-Wentrup F. (2015). TLR3 Triggering Regulates PD-L1 (CD274) Expression in Human Neuroblastoma Cells. Cancer Lett..

[91] Qin Z., Zhang L., Xu Y., Zhang X., Fang X., Qian D., Liu X., Liu T., Li L., Yu H. (2018). TLR3 Regulates PD-L1 Expression in Human Cytomegalovirus Infected Glioblastoma. Int. J. Clin. Exp. Pathol..

[92] Montani M.S.G., Santarelli R., Falcinelli L., Gonnella R., Granato M., Di Renzo L., Cuomo L., Vitillo M., Faggioni A., Cirone M. (2018). EBV Up-Regulates PD-L1 on the Surface of Primary Monocytes by Increasing ROS and Activating TLR Signaling and STAT3. J. Leukoc. Biol..

[93] Beswick E.J., Johnson J.R., Saada J.I., Humen M., House J., Dann S., Qiu S., Brasier A.R., Powell D.W., Reyes V.E. (2014). TLR4 Activation Enhances the PD-L1–Mediated Tolerogenic Capacity of Colonic CD90+ Stromal Cells. J. Immunol..

[94] Chi D., Xu W., Tao X., Zhang T., Cui Y. (2020). PD-L1 Expression in Colorectal Cancer and Its Relationship with TLR-4 Expression. J. BUON.

[95] Barsoum I.B., Smallwood C.A., Siemens D.R., Graham C.H. (2014). A Mechanism of Hypoxia-Mediated Escape from Adaptive Immunity in Cancer Cells. Cancer Res..

[96] Noman M.Z., Desantis G., Janji B., Hasmim M., Karray S., Dessen P., Bronte V., Chouaib S. (2014). PD-L1 Is a Novel Direct Target of HIF-1α, and Its Blockade under Hypoxia Enhanced MDSC-Mediated T Cell Activation. J. Exp. Med..

[97] Ding X., Wang L., Zhang X., Xu J., Li P., Liang H., Zhang X., Xie L., Zhou Z., Yang J. (2021). The Relationship between Expression of PD-L1 and HIF-1α in Glioma Cells under Hypoxia. J. Hematol. Oncol..

[98] Chen Z.-Q., Zuo X.-L., Cai J., Zhang Y., Han G.-Y., Zhang L., Ding W.-Z., Wu J.-D., Wang X.-H. (2023). Hypoxia-Associated CircPRDM4 Promotes Immune Escape via HIF-1α Regulation of PD-L1 in Hepatocellular Carcinoma. Exp. Hematol. Oncol..

[99] Yin S., Guo Y., Wen X., Zeng H., Chen G. (2022). Increased Expression of PD-L1 in Endometrial Cancer Stem-like Cells Is Regulated by Hypoxia. Front. Biosci. (Landmark Ed.).

[100] Van Duijn A., Willemsen K.J., van Uden N.O.P., Hoyng L., Erades S., Koster J., Luiten R.M., Bakker W.J. (2022). A Secondary Role for Hypoxia and HIF1 in the Regulation of (IFNγ-Induced) PD-L1 Expression in Melanoma. Cancer Immunol. Immunother..

[101] Smith V., Mukherjee D., Lunj S., Choudhury A., Hoskin P., West C., Illidge T. (2021). The Effect of Hypoxia on PD-L1 Expression in Bladder Cancer. BMC Cancer.

[102] Petridou S., Maltseva O., Spanakis S., Masur S.K. (2000). TGF-beta receptor expression and smad2 localization are cell density dependent in fibroblasts. Investig. Ophthalmol. Vis. Sci..

[103] Pocsik E., Mihalik R., Ali-Osman F., Aggarwal B.B. (1994). Cell density-dependent regulation of cell surface expression of two types of human tumor necrosis factor receptors and its effect on cellular response. J Cell. Biochem..

[104] Quan Z., Yang Y., Zheng H., Zhan Y., Luo J., Ning Y., Fan S. (2022). Clinical Implications of the Interaction between PD-1/PD-L1 and PI3K/AKT/MTOR Pathway in Progression and Treatment of Non-Small Cell Lung Cancer. J. Cancer.

[105] Baretić D., Williams R.L. (2014). PIKKs—The Solenoid Nest Where Partners and Kinases Meet. Curr. Opin. Struct. Biol..

[106] Sengupta S., Peterson T.R., Sabatini D.M. (2010). Regulation of the MTOR Complex 1 Pathway by Nutrients, Growth Factors, and Stress. Mol. Cell.

[107] Kim Y.C., Guan K.-L. (2015). MTOR: A Pharmacologic Target for Autophagy Regulation. J. Clin. Investig..

[108] Liu P., Gan W., Chin Y.R., Ogura K., Guo J., Zhang J., Wang B., Blenis J., Cantley L.C., Toker A. (2015). PtdIns(3,4,5)P3-Dependent Activation of the MTORC2 Kinase Complex. Cancer Discov..

[109] Zinzalla V., Stracka D., Oppliger W., Hall M.N. (2011). Activation of MTORC2 by Association with the Ribosome. Cell.

[110] Tian T., Li X., Zhang J. (2019). MTOR Signaling in Cancer and MTOR Inhibitors in Solid Tumor Targeting Therapy. Int. J. Mol. Sci..

[111] Koh V., Chakrabarti J., Torvund M., Steele N., Hawkins J.A., Ito Y., Wang J., Helmrath M.A., Merchant J.L., Ahmed S.A. (2021). Hedgehog Transcriptional Effector GLI Mediates MTOR-Induced PD-L1 Expression in Gastric Cancer Organoids. Cancer Lett..

[112] Lastwika K.J., Wilson W., Li Q.K., Norris J., Xu H., Ghazarian S.R., Kitagawa H., Kawabata S., Taube J.M., Yao S. (2016). Control of PD-L1 Expression by Oncogenic Activation of the AKT–MTOR Pathway in Non–Small Cell Lung Cancer. Cancer Res..

[113] Mansour F.A., Al-Mazrou A., Al-Mohanna F., Al-Alwan M., Ghebeh H. (2020). PD-L1 Is Overexpressed on Breast Cancer Stem Cells through Notch3/MTOR Axis. OncoImmunology.

[114] Tong G., Cheng B., Li J., Wu X., Nong Q., He L., Li X., Li L., Wang S. (2019). MACC1 Regulates PDL1 Expression and Tumor Immunity through the C-Met/AKT/MTOR Pathway in Gastric Cancer Cells. Cancer Med..

[115] Zhang Q., Zhang Y., Chen Y., Qian J., Zhang X., Yu K. (2019). A novel mTORC1/2 inhibitor (MTI-31) inhibits tumor growth, epithelial-mesenchymal transition, metastases, and improves antitumor immunity in preclinical models of lung Cancer. Clin. Cancer Res..

[116] Zhang D., Reyes R.M., Osta E., Kari S., Gupta H.B., Padron A.S., Kornepati A.V.R., Kancharla A., Sun X., Deng Y. (2021). Bladder Cancer Cell-Intrinsic PD-L1 Signals Promote MTOR and Autophagy Activation That Can Be Inhibited to Improve Cytotoxic Chemotherapy. Cancer Med..

[117] Deng L., Qian G., Zhang S., Zheng H., Fan S., Lesinski G.B., Owonikoko T.K., Ramalingam S.S., Sun S.Y. (2019). Inhibition of MTOR Complex 1/P70 S6 Kinase Signaling Elevates PD-L1 Levels in Human Cancer Cells through Enhancing Protein Stabilization Accompanied with Enhanced β-TrCP Degradation. Oncogene.

[118] Zhang C., Duan Y., Xia M., Dong Y., Chen Y., Zheng L., Chai S., Zhang Q., Wei Z., Liu N. (2019). TFEB Mediates Immune Evasion and Resistance to MTOR Inhibition of Renal Cell Carcinoma via Induction of PD-L1. Clin. Cancer Res..

[119] Hirayama Y., Gi M., Yamano S., Tachibana H., Okuno T., Tamada S., Nakatani T., Wanibuchi H. (2016). Anti-PD-L1 Treatment Enhances Antitumor Effect of Everolimus in a Mouse Model of Renal Cell Carcinoma. Cancer Sci..

[120] Wang C., Yi T., Qin L., Maldonado R.A., von Andrian U.H., Kulkarni S., Tellides G., Pober J.S. (2013). Rapamycin-Treated Human Endothelial Cells Preferentially Activate Allogeneic Regulatory T Cells. J. Clin. Investig..

[121] Azuma T., Yao S., Zhu G., Flies A.S., Flies S.J., Chen L. (2008). B7-H1 Is a Ubiquitous Antiapoptotic Receptor on Cancer Cells. Blood.

[122] Li J., Chen L., Xiong Y., Zheng X., Xie Q., Zhou Q., Shi L., Wu C., Jiang J., Wang H. (2017). Knockdown of PD-L1 in Human Gastric Cancer Cells Inhibits Tumor Progression and Improves the Cytotoxic Sensitivity to CIK Therapy. Cell. Physiol. Biochem..

[123] Lou Y., Diao L., Cuentas E.R.P., Denning W.L., Chen L., Fan Y.H., Byers L.A., Wang J., Papadimitrakopoulou V.A., Behrens C. (2016). Epithelial–Mesenchymal Transition Is Associated with a Distinct Tumor Microenvironment Including Elevation of Inflammatory Signals and Multiple Immune Checkpoints in Lung Adenocarcinoma. Clin. Cancer Res..

[124] Alsuliman A., Colak D., Al-Harazi O., Fitwi H., Tulbah A., Al-Tweigeri T., Al-Alwan M., Ghebeh H. (2015). Bidirectional Crosstalk between PD-L1 Expression and Epithelial to Mesenchymal Transition: Significance in Claudin-Low Breast Cancer Cells. Mol. Cancer.

[125] Aghajani M.J., Yang T., Schmitz U., James A., McCafferty C.E., de Souza P., Niles N., Roberts T.L. (2020). Epithelial-to-Mesenchymal Transition and Its Association with PD-L1 and CD8 in Thyroid Cancer. Endocr. Connect..

[126] Gong X., Li X., Jiang T., Xie H., Zhu Z., Zhou F., Zhou C. (2017). Combined Radiotherapy and Anti–PD-L1 Antibody Synergistically Enhances Antitumor Effect in Non–Small Cell Lung Cancer. J. Thorac. Oncol..

[127] Tu X., Qin B., Zhang Y., Zhang C., Kahila M., Nowsheen S., Yin P., Yuan J., Pei H., Li H. (2019). PD-L1 (B7-H1) Competes with the RNA Exosome to Regulate the DNA Damage Response and Can Be Targeted to Sensitize to Radiation or Chemotherapy. Mol. Cell.

[128] Chang C.-H., Qiu J., O’Sullivan D., Buck M.D., Noguchi T., Curtis J.D., Chen Q., Gindin M., Gubin M.M., van der Windt G.J.W. (2015). Metabolic Competition in the Tumor Microenvironment Is a Driver of Cancer Progression. Cell.

[129] Schulz D., Streller M., Piendl G., Brockhoff G., Reichert T.E., Menevse A.N., Beckhove P., Hautmann M.G., Bauer R.J., Ettl T. (2020). Differential Localization of PD-L1 and Akt-1 Involvement in Radioresistant and Radiosensitive Cell Lines of Head and Neck Squamous Cell Carcinoma. Carcinogenesis.

[130] Clark C.A., Gupta H.B., Sareddy G., Pandeswara S., Lao S., Yuan B., Drerup J.M., Padron A., Conejo-Garcia J., Murthy K. (2016). Tumor-Intrinsic PD-L1 Signals Regulate Cell Growth, Pathogenesis, and Autophagy in Ovarian Cancer and Melanoma. Cancer Res..

[131] Gupta H.B., Clark C.A., Yuan B., Sareddy G., Pandeswara S., Padron A.S., Hurez V., Conejo-Garcia J., Vadlamudi R., Li R. (2016). Tumor Cell-Intrinsic PD-L1 Promotes Tumor-Initiating Cell Generation and Functions in Melanoma and Ovarian Cancer. Signal Transduct. Target. Ther..

[132] Wang F., Yang L., Xiao M., Zhang Z., Shen J., Anuchapreeda S., Tima S., Chiampanichayakul S., Xiao Z. (2022). PD-L1 Regulates Cell Proliferation and Apoptosis in Acute Myeloid Leukemia by Activating PI3K-AKT Signaling Pathway. Sci. Rep..

[133] Cui P., Jing P., Liu X., Xu W. (2020). Prognostic Significance of PD-L1 Expression and Its Tumor-Intrinsic Functions in Hypopharyngeal Squamous Cell Carcinoma. Cancer Manag. Res..

[134] Gato-Cañas M., Zuazo M., Arasanz H., Ibañez-Vea M., Lorenzo L., Fernandez-Hinojal G., Vera R., Smerdou C., Martisova E., Arozarena I. (2017). PDL1 Signals through Conserved Sequence Motifs to Overcome Interferon-Mediated Cytotoxicity. Cell Rep..

[135] Wang X.R., Jiang Z.B., Xu C., Meng W.Y., Liu P., Zhang Y.Z., Xie C., Xu J.Y., Xie Y.J., Liang T.L. (2022). Andrographolide Suppresses Non-Small-Cell Lung Cancer Progression through Induction of Autophagy and Antitumor Immune Response. Pharmacol. Res..

[136] Xie C., Zhou X., Liang C., Li X., Ge M., Chen Y., Yin J., Zhu J., Zhong C. (2021). Apatinib Triggers Autophagic and Apoptotic Cell Death via VEGFR2/STAT3/PD-L1 and ROS/Nrf2/P62 Signaling in Lung Cancer. J. Exp. Clin. Cancer Res..

[137] Zhu J., Li Y., Luo Y., Xu J., Liufu H., Tian Z., Huang C., Li J., Huang C. (2019). A Feedback Loop Formed by ATG7/Autophagy, FOXO3a/MiR-145 and PD-L1 Regulates Stem-Like Properties and Invasion in Human Bladder Cancer. Cancers.

[138] Park S.S., Kim J.I., Lee C.H., Bae J.H., Park J.M., Choe E.J., Baek M.C. (2022). Temsirolimus Enhances Anti-Cancer Immunity by Inducing Autophagy-Mediated Degradation of the Secretion of Small Extracellular Vesicle PD-L1. Cancers.

[139] Wang Y., Zhang H., Qin Z.H. (2019). Regulation of Autophagy by MTOR Signaling Pathway. Autophagy: Biology and Diseases: Basic Science.

[140] Clark C.A., Gupta H.B., Curiel T.J. (2017). Tumor Cell-Intrinsic CD274/PD-L1: A Novel Metabolic Balancing Act with Clinical Potential. Autophagy.

[141] Spirina L., Avgustinovich A., Afanas’ev S., Volkov M., Dobrodeev A., Cheremisina O., Kostromitsky D. (2021). PD-L1 Status in Gastric Cancers, Association with the Transcriptional, Growth Factors, AKT/MTOR Components Change, and Autophagy Initiation. Int. J. Mol. Sci..

[142] Chen R.Q., Xu X.H., Liu F., Li C.Y., Li Y.J., Li X.R., Jiang G.Y., Hu F., Liu D., Pan F. (2019). The Binding of PD-L1 and Akt Facilitates Glioma Cell Invasion Upon Starvation via Akt/Autophagy/F-Actin Signaling. Front. Oncol..

[143] Chen Z., Liu S., Xie P., Zhang B., Yu M., Yan J., Jin L., Zhang W., Zhou B., Li X. (2022). Tumor-Derived PD1 and PD-L1 Could Promote Hepatocellular Carcinoma Growth through Autophagy Induction in Vitro. Biochem. Biophys. Res. Commun..

